# The effect of calcium electroporation on viability, phenotype and function of melanoma conditioned macrophages

**DOI:** 10.1038/s41598-020-77743-2

**Published:** 2020-11-26

**Authors:** Liam Friel Tremble, Cynthia C. B. B. Heffron, Patrick F. Forde

**Affiliations:** 1 CancerResearch@UCC, University College Cork, Fourth floor, Western Gateway Building, Western Road, Cork, Ireland; 2 Department of Pathology, Cork University Hospital, Wilton, Cork, Ireland

**Keywords:** Cancer, Cancer microenvironment

## Abstract

Electroporation in combination with chemotherapy is an established treatment used on solid malignancies that results in enhanced chemotherapeutic uptake. Recent advances have begun to transition to the use of non-toxic compounds, such as calcium, in lieu of chemotherapy, which can also induce tumour cell death. While the effect of treatment on tumour cell death has been well characterized and has been shown to induce an immunogenic form of cell death, the effect of treatment on intratumoural immune cells has not been investigated. Here we present data showing the effect of calcium electroporation on immune cells, using melanoma-conditioned bone marrow-derived macrophages. Similar to tumour cells, macrophage cell membranes are susceptible to poration following treatment and subsequently reseal. Macrophages are less susceptible to calcium electroporation induced cell death in comparison to B16F10 melanoma cells. However treatment with electroporation with or without bleomycin or calcium was shown to affect macrophage phenotype and function. Coculture of calcium electroporated macrophages revealed that both the capacity of macrophages to stimulate and direct T cell responses are affected following exposure to treatment. We conclude that calcium electroporation has the potential to boost the immunogenic capacity of exposed tumour associated macrophages, and further research is warranted to determine if calcium electroporation can be optimised to generate systemic anti-cancer immune responses.

## Introduction

Reversible electroporation (EP) is a local treatment modality which can be used to form transient pores in the cell membrane^1,2^. Electrodes are placed in direct proximity to the tissue of interest and a series of pulses are delivered which result in the formation of pores which can persist for several seconds to several minutes before resealing and leaving cells intact and viable^3^.

Reversible EP has been used in a cancer context to increase the uptake of chemotherapeutics, especially hydrophobic drugs, such as bleomycin, which can have poor rates of passive cell penetration^4–6^. However, EP has also been used in combination with non-toxic drugs such as calcium, which when delivered in sufficient concentrations can induce rapid cell death^7^. Due to variations in cell turnover rates, EP can be safely used in proximity to many healthy tissues and vasculature, making it an attractive alternative in a range of tumour types. Previous studies have shown that while microvasculature can be damaged, vasculature with a diameter of 5 mm or more are preserved during treatment, while the collagen matrix scaffolding of the liver has also been unaffected by treatment^8–10^.

EP with chemotherapy, termed electrochemotherapy (ECT), is routinely used for the treatment of dermal lesions and endoscopic devices have been designed for the treatment of oesophageal and colorectal cancers^11–15^. Other devices are currently in development to facilitate EP of other organ sites^16^. Complete response rates in excess of 80% have regularly been reported for treatment of dermal lesions with ECT^17^.

ECT with bleomycin has been shown to induce an immunogenic form of cell death characterized by enhanced ecto-calreticulin and ATP and HMGB1 release by treated cells^18^. An influx of immune cells is also seen following treatment, including macrophages, indicating that ECT may be utilized to induce an anti-cancer immune response^19^. However, while ECT has been shown to repress distal tumour growth in murine models, this is not seen in clinically advanced cancer patients^19,20^.

The substitution of chemotherapy with calcium was rationally designed in part due to the hypothesized immunogenic effects of calcium uptake, and to abrogate the immunosuppressive effects of chemotherapy. Promisingly, a case study in malignant melanoma treated with calcium EP has reported remission of both local treated and distal untreated tumours, indicating a systemic anti-cancer immune response or other abscopal effect^7^.

Due to the immunogenic potential of EP treatments, the effect of treatment in combination with other immunogenic modalities, including T cell check point inhibitors, is under investigation^21,22^. However the direct effect of EP on intratumoural immune cells is not known. We aimed to determine if tumour-associated macrophages (TAMs) are sensitive to ECT or calcium EP and to determine the immunogenic capacity of TAMs following exposure to calcium EP.

Here, using a bone marrow-derived monocyte (BMDM) model of melanoma TAMs, we show for the first time that EP can induce reversible pore formation on BMDMs using pulses optimised for reversible EP of tumour cells derived from the European Standard Operating Procedures of ECT (ESOPE) study^4^. Using these pulses we have shown that treatment can impact BMDM size, viability, phenotype, function and role in T cell activation.

## Results

### Optimization of B16F10 EP parameters

**Figure 1:**
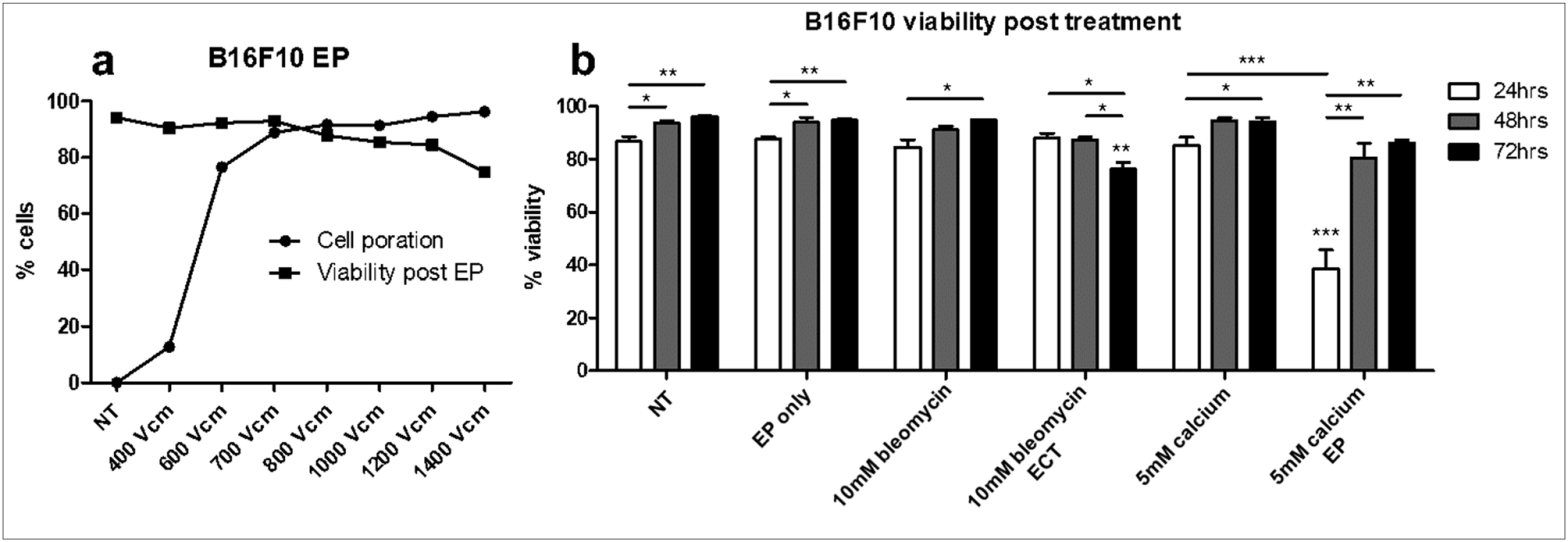
Optimisation of electroporation parameters in B16F10 cells. B16F10 cells were electroporated at a range of concentrations and cell poration was measured by PI uptake during EP (**a**). Cell viability was determined by PI permeability 30 min following treatment. Cells were electroporated at 700 V/cm with or without drug and were analysed for viability 24, 48 and 72 h post treatment (**b**). Data shown is representative of an n = 3 per group. *p < 0.05, **p < 0.01, ***p < 0.001 compared to NT unless specified.

As electrical parameters of tumour EP are optimized to induce reversible pore formation on tumour cells, we first optimized the in vitro delivery of EP on B16F10 tumour cells^23–25^. Cell poration was measured in parallel with the subsequent viability of cells 30 min following EP (Fig. 1a). At 800 V/cm the sum of non-viable cells and porated cells surpassed 100%, indicating that a proportion of electroporated cells did not regain membrane integrity. As such 700 V/cm was selected as an appropriate voltage to induce reversible EP in B16F10 cells.

Using this voltage preliminary data was generated to ensure cell viability at 24, 48, and 72 h post treatment (Fig. 1b). Using previously optimised concentrations of bleomycin (10 nM) and calcium (0.5–5 mM) we examined the nature of cell death following treatment. No loss in viability was observed following EP. A daily increase in viability was observed in untreated cells, however this was considered likely to be an artefact of experimental technique, in which B16F10 cells do not respond optimally to repeated trypsinisations within short periods and the nature of the EP buffer used.

Significantly decreased viability was seen in cells treated with bleomycin ECT 3 days post treatment compared to EP only (*p* < 0.01) and in contrast a significant decrease was seen in 24 h post treatment in cells treated with calcium EP (*p* < 0.001), which was no longer apparent at 48 or 72 h. As expected these results indicated a differential timing of cell death following EP with calcium and bleomycin.

### Cell death following treatment of B16F10s

**Figure 2:**
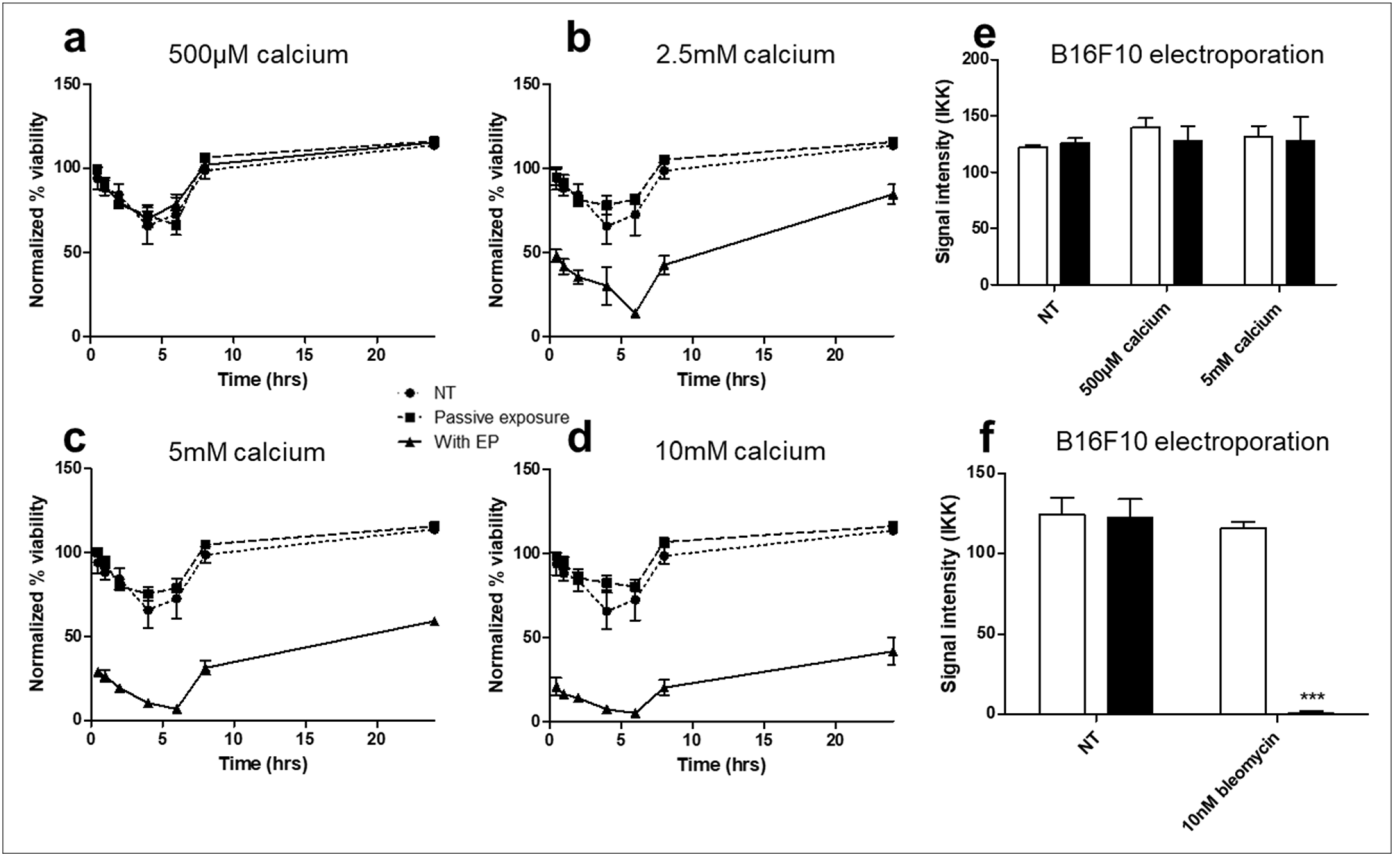
Cell death following electroporation with bleomycin or calcium. B16F10 cells were electroporated at 700 V/cm with or without drug. Serial matched measurements of cell viability were taken following calcium EP (**a**–**d**). Viability was normalized to untreated cells a t = 0. Viable cells were isolated 24 h post treatment by selection of adherent cells and seeded at a low concentration for clonogenic analysis (**e**,**f**). Data shown is representative of an n = 3 per group (**a**–**d**,**f**) and an n = 9 per group (**e**). ***p < 0.001 compared to EP only.

To further determine the nature of cell death following treatment, we performed serial matched viability analysis on B16F10 cells following calcium EP at a range of calcium concentrations (Fig. 2a–d). No cell death was observed in cells treated with 500 μM calcium, indicating doses of 2.5 mM or greater are required to generate therapeutic cell death in a B16F10 model of melanoma. A significant drop in viability was seen in cells treated with 2.5 mM calcium or higher in combination with EP, which had occurred by 30 min post treatment. Cell death increased until 6 h after treatment, but recovery was seen in all conditions by 8 h. The doubling time of B16F10s is approximately 15 h, so improved viability is likely a result of replication by intact cells rather than the recovery of cells which had lost membrane integrity. For further studies we chose to use 500 μM as a low ineffective dose and 5 mM as the upper dose as maximal death percentages between 5 and 10 mM were non-significant.

To determine if cells not acutely killed following calcium EP had any loss in malignant potential, we performed clonogenic assays on cells which were viable 24 h following treatment (Fig. 2e). No change was seen in clonogenic ability, indicating that cells not killed acutely have normal replicative potential.

Conversely, we performed clonogenics with viable cells selected 24 h following EP with bleomycin (Fig. 2f). No colonies were established in comparison to all controls (*p* < 0.001), in concordance with previous literature indicating that ECT results in cell death by loss of replicative potential.

### Melanoma conditioned BMDM membrane poration and size following EP

To determine the effect of tumour EP on intratumoural macrophages, melanoma conditioned BMDMs were electroporated with the parameters determined in Fig. 1.

**Figure 3:**
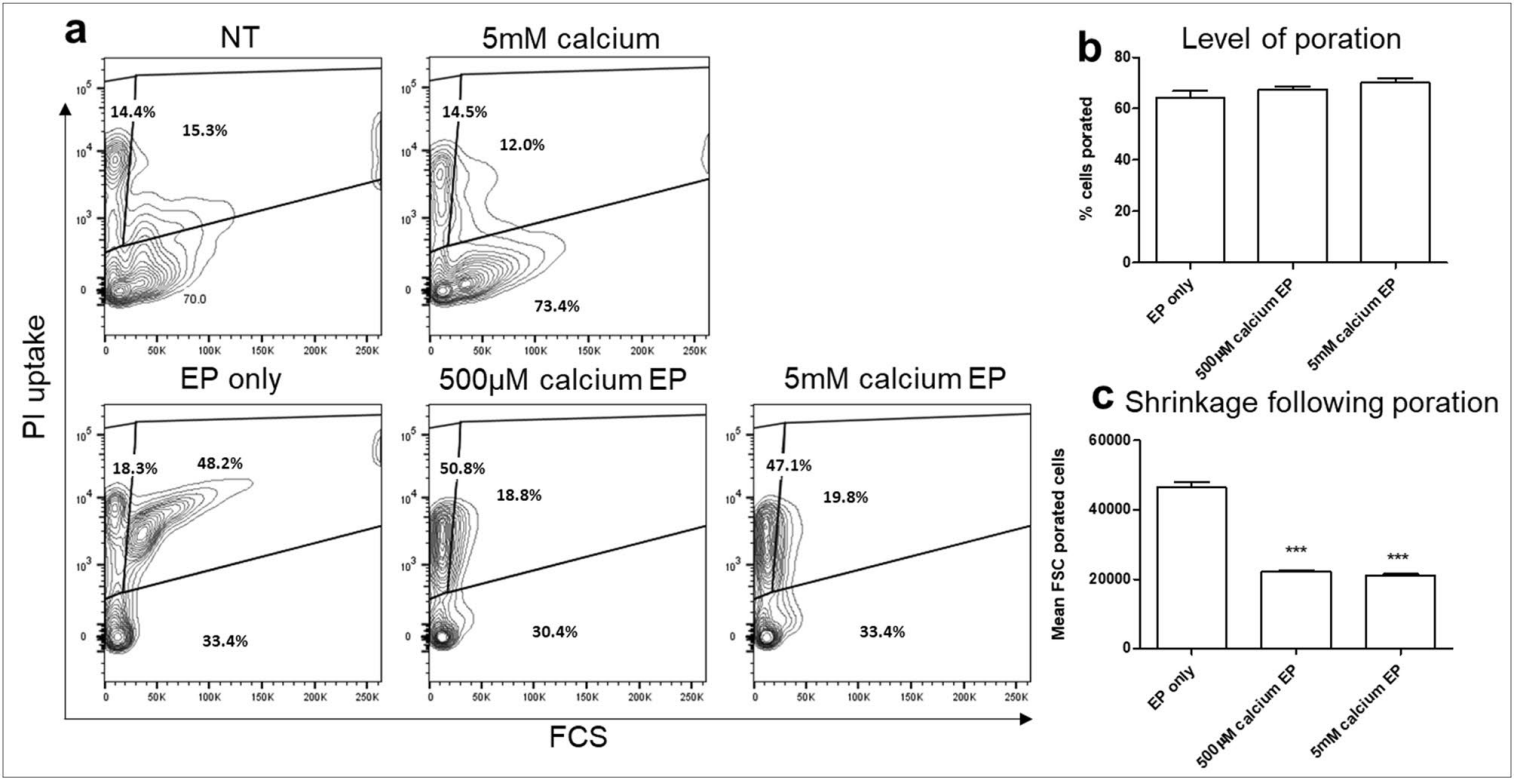
Electroporation of melanoma conditioned BMDMs. Melanoma conditioned BMDMs were electroporated at the same parameters as used for B16F10 cells. Poration was measured by PI uptake during EP (**a**,**b**). Cell size was determined by flow cytometric forward scatter readings (**c**). Data shown is representative of an n = 3 per group. ***p < 0.001 compared to EP only.

Calcium is a positively charged molecule (Ca^2+^), which contributes to the membrane potential of the plasma membrane by virtue of the different concentrations of free calcium inside and outside of the cell. Intracellular calcium concentrations are tightly regulated to facilitate its role as a secondary signalling messenger, thus increased extracellular concentrations result in higher voltage gradients being formed across the plasma membrane. It has previously been shown that calcium can affect membrane resealing and enhance the rate of tumour cell death following EP, both in vitro and in vivo^26,27^. Thus, The efficiency of EP in the presence of calcium was also investigated as extracellular calcium is known to affect membrane resealing and other membrane dynamics through its role in voltage generation (Fig. 3a)^28^.

EP induced cell poration in 64 ± 5% of BMDMs, which was unchanged by the addition of calcium (Fig. 3b).

Although calcium did not affect the level of porated cells, there was a shift in cell size following EP, with calcium EP resulting in phenotypically smaller cells as determined by forward scatter (*p* < 0.001) (Fig. 3a,c). This effect was induced by both 5 mM calcium and the lower dose 500 μM which was non-lethal in B16F10 cells. The reduction in FSC may indicate that larger BMDMs are more susceptible to EP.

### Melanoma conditioned BMDM viability following EP

**Figure 4:**
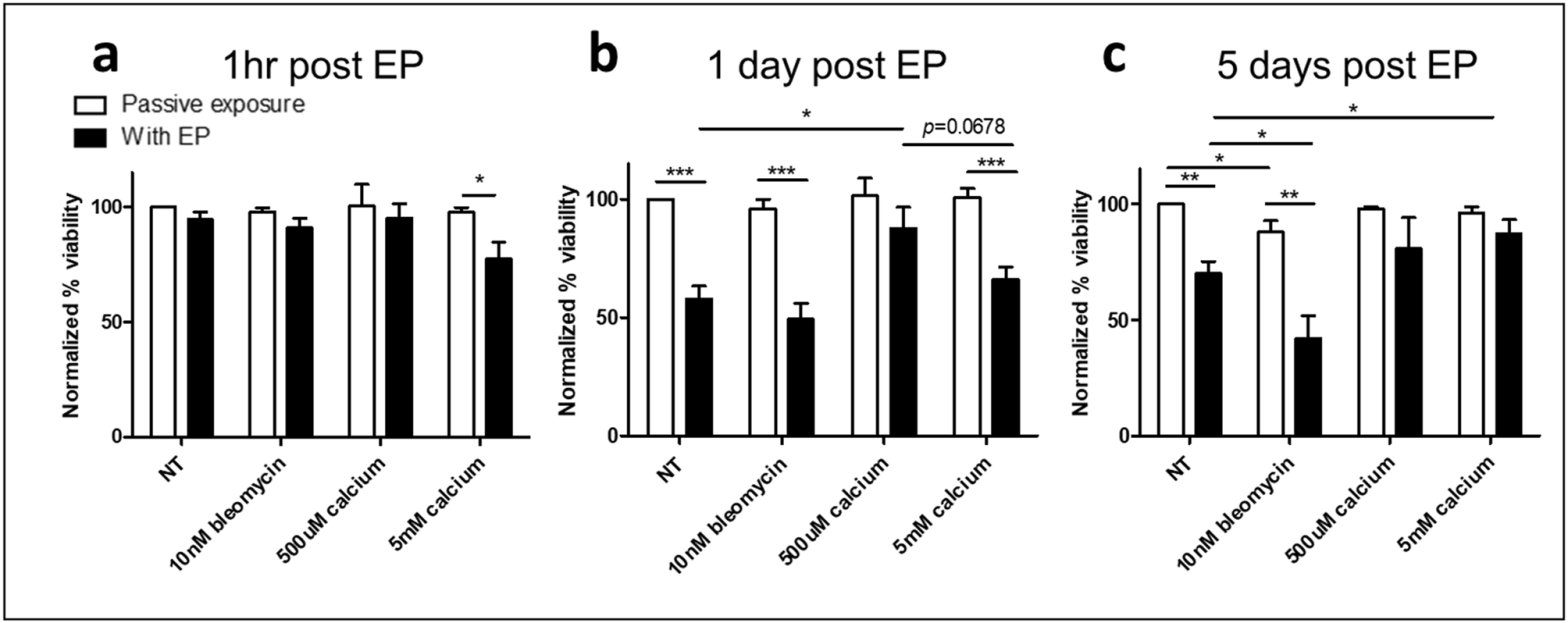
Viability of melanoma conditioned BMDMs following electroporation with bleomycin or calcium. Melanoma conditioned BMDMs were electroporated in the presence of bleomycin or calcium. Viability was determined by PI uptake at 1 h (**a**), 1 day (**b**) and 5 days (**c**) post treatment. Data shown is representative of an n = 7 per group and normalized to untreated controls. *p < 0.05, **p < 0.01, ***p < 0.001 compared to NT or EP only as appropriate unless specified.

Following determination that BMDMs are porated, we next analysed the viability of cells following treatment with EP with or without calcium or bleomycin (Fig. 4). As BMDMs have limited replicative potential the effect of therapy was followed for up to 5 days. At 1 h post treatment, all cells except those treated with EP with 5 mM calcium (*p* < 0.05) were as viable as untreated controls (Fig. 4a). These results indicate that following poration, in which over 60% of cells are porated, cells regain membrane integrity.

At 24 h post treatment, cells treated with EP with or without bleomycin displayed a marked reduction in viability, indicating an EP related toxicity in BMDMs (Fig. 4b). This is likely due to changes in the composition of their cytoplasmic contents which can be distorted by loss of cytoplasm and gain of extracellular medium during poration. 500 μM calcium was protective to electroporated cells, resulting in significantly reduced death compared to EP alone (*p* < 0.05). There was no significant difference between 500 μM calcium EP and untreated cells although there was a slight trend of decreased viability. This protective role was lost in the presence of 5 mM calcium (*p* = 0.0678). Thus low levels of calcium are most protective for conditioned BMDMs following treatment.

At 5 days post treatment the decrease in viability of electroporated cells was less pronounced but still present (*p* < 0.01, Fig. 4c). The addition of bleomycin decreased the viability of untreated and electroporated cells, however bleomycin induced a larger level of cell death in combination with EP compared to passive exposure (30 ± 13% vs 46 ± 16%, *p* = 0.0642). Both doses of calcium proved protective at 5 days post treatment, with no significant change from untreated or passive calcium exposure and increased viability compared to EP alone (*p* < 0.05).

### Melanoma conditioned BMDM phenotypic shift following EP

**Figure 5:**
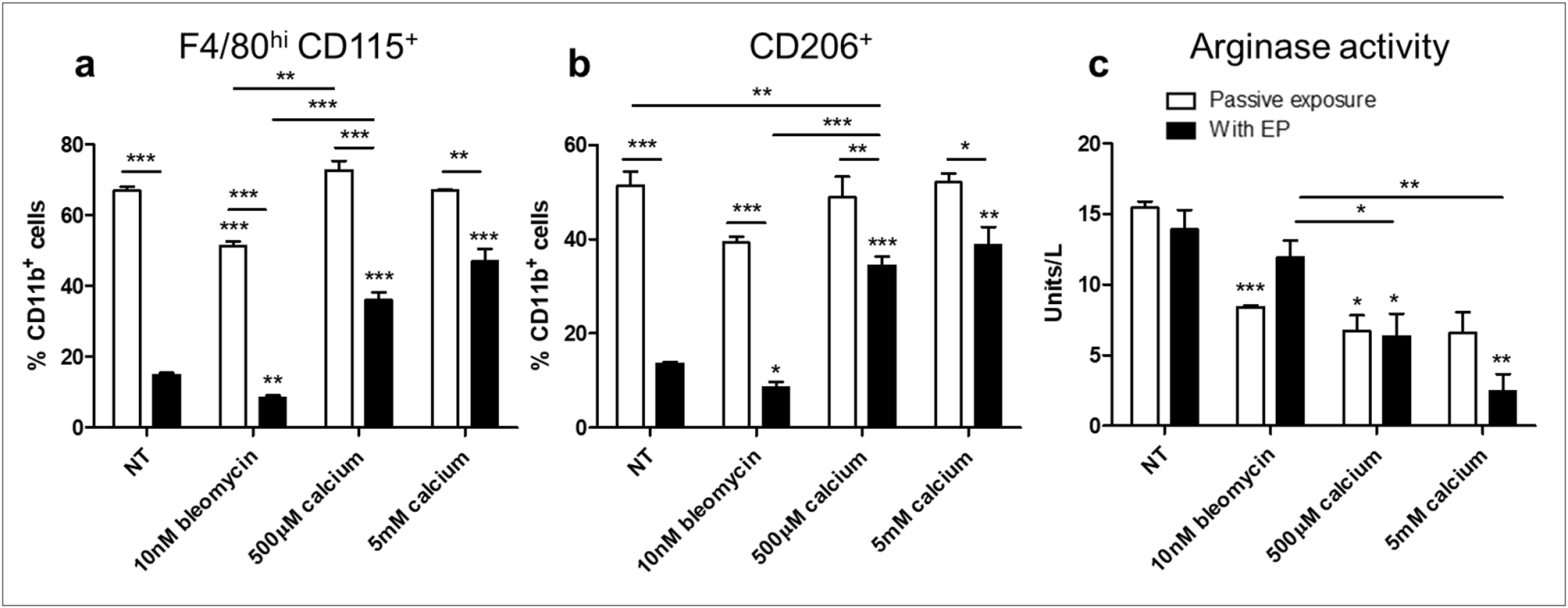
Phenotypic evaluation of melanoma conditioned BMDMs following electroporation with bleomycin or calcium. Melanoma conditioned BMDMs were electroporated in the presence of bleomycin or calcium and cultured for 72 h. Cells were analysed by flow cytometry (**a**,**b**) for the macrophage activation markers F4/80, CD115 and CD206. BMDMs were identified as CD11b^+^ and gated according to F4/80, CD115 and CD206 expression. Intracellular arginase activity was assayed following cell lysis (**c**). Data shown is representative of an n = 3 per group. *p < 0.05, **p < 0.01, ***p < 0.001 compared to NT or EP only as appropriate unless specified.

Following confirmation that a proportion of BMDMs can survive EP, we next sough to phenotype cells to determine any shifts in behaviour. Cells were isolated 72 h after treatment and the canonical M1:M2 enzymes, iNOS and arginase, were assayed in combination with cell surface markers. Viable CD11b^+^ BMDMs can be separated in a binary fashion into F4/80^+^ CD115^lo^ or F4/80^hi^ CD115^+^ cells. An upregulation of F4/80 accompanied with CD115 upregulation is indicative in murine myeloid cells of a transition from monocyte to macrophage^29^. The linear evolution of these cells is poorly understood, however this shift is also associated with an upregulation of M2-like behaviour^30^. EP resulted in a significant decrease in this maturation process (Fig. 5a), which was maintained in cells treated with bleomycin EP. However the addition of calcium, in a dose dependent manner, reduced this decrease (*p* < 0.001). The M2-like phenotype of these matured cells was reflected by the CD206 expression of CD11b^+^ cells (Fig. 5b).

All cells assayed were negative for detectable nitrates in supernatant using a Griess assay (data not shown). While EP induced a decrease in F4/80^hi^ CD115^+^ cells, there was no change in arginase activity in these cells (Fig. 5c). Despite a relative increase in F4/80^hi^ CD115^+^ in cells treated with calcium EP, these cells displayed marked reduction in arginase activity, that was independent of EP, indicating that passive exposure to calcium can induce metabolic effects in BMDMs.

### Effect of BMDM calcium EP on cocultured CD4^+^ T cells

Due to enhanced survival of cells treated with calcium EP, and increased maturation coupled with decreased arginase activity this treatment was selected as the most promising for further immunological studies. The effect of treated BMDMs on CD4^+^ T cells was determined by coculture experiments using BMDMs isolated 24 h after treatment in combination with freshly isolated splenic CD4^+^ T cells.

**Figure 6:**
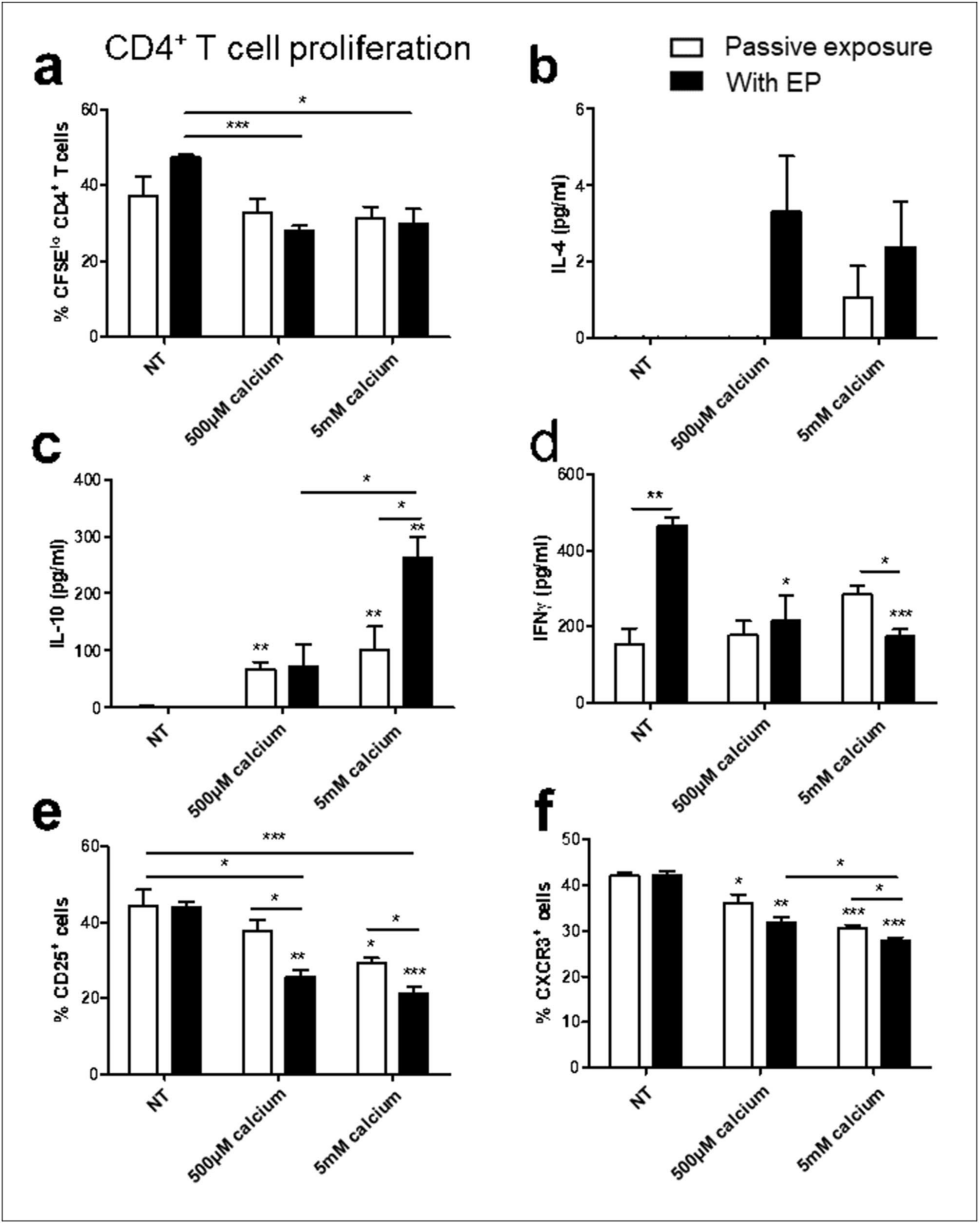
Effect of melanoma conditioned BMDMs following electroporation on CD4^+^ T cells. Melanoma conditioned BMDMs were electroporated in the presence of bleomycin or calcium and cultured for 24 h. Cells were then cocultured with freshly isolated CFSE labelled CD4^+^ T cells. T cell proliferation was measured by reduced CFSE expression (**a**). Levels of IL-4 (**b**), IL-10 (**c**) and IFNγ (**d**) in the supernatant were measured by ELISA. Activated T cells were gated by low CFSE expression and CD25 (**e**) and CXCR3 (**f**) expression was determined by flow cytometry. Data shown is representative of an n = 3 per group. *p < 0.05, **p < 0.01, ***p < 0.001 compared to NT or EP only as appropriate unless specified.

Calcium EP of BMDMs was found to decrease their ability to stimulate CD4^+^ T cell proliferation (Fig. 6a). Cells treated with or without EP resulted in no detectable IL-10 in supernatant (Fig. 6c), however calcium treatment induced IL-10 secretion. This effect was further increased by 5 mM calcium in combination with EP (*p* < 0.05). IFNγ secretion patterns inversely reflected IL-10 expression (Fig. 6d). EP alone induced high levels, and the addition of calcium reduced IFNγ secretion. These results suggest that while EP alone may promote relative Th1 responses, calcium EP shifts this towards a regulatory T cell (Treg) response. This is also reflected by the Th2 cytokine IL-4, which often accompanies Treg responses, and which was detectable in trace amounts following treatment with calcium EP (Fig. 6b).

A decrease in IFNγ was reflected by decreased CXCR3 expression on activated T cells (Fig. 6f) following calcium EP. There was a decrease also in the Treg marker, CD25, following calcium EP (*p* < 0.01), however CD25 expression is also coupled to T cell activation and could reflected decreased activation as is mirrored in the reduced T cell proliferation seen in Fig. 6a.

### Effect of BMDM calcium EP on cocultured CD8^+^ T cells

**Figure 7:**
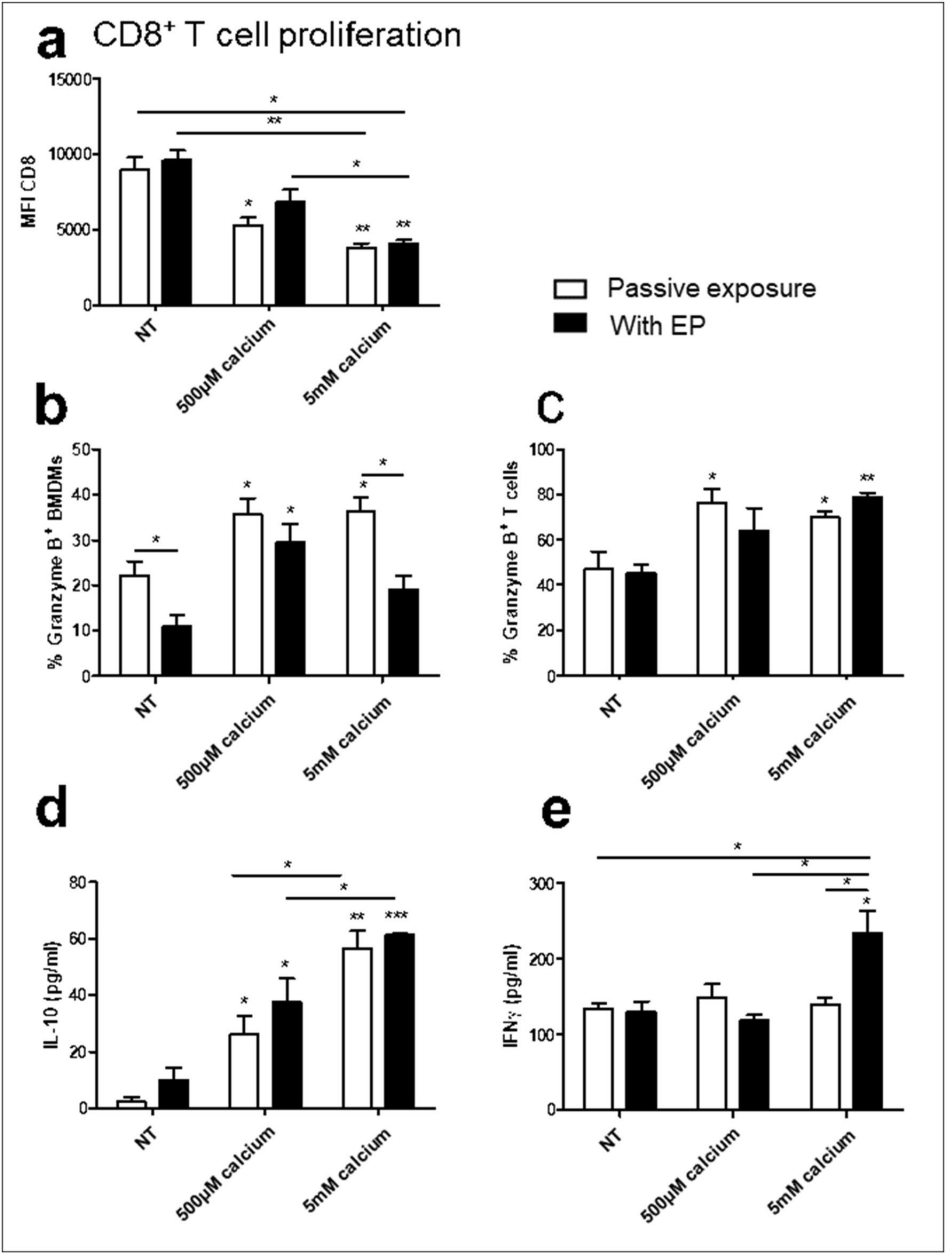
Effect of melanoma conditioned BMDMs following electroporation on CD8^+^ T cells. Melanoma conditioned BMDMs were electroporated in the presence of bleomycin or calcium and cultured for 24 h. Cells were then cocultured with freshly isolated CFSE labelled CD8^+^ T cells. T cell proliferation was measured by reduced CFSE expression (**a**). Intracellular levels of granzyme B were determined by flow cytometry in treated BMDMs (**b**) and cocultured activated CD8^+^ T cells (**c**). Levels of IL-10 (**d**) and IFNγ (**e**) in the supernatant were measured by ELISA. Data shown is representative of an n = 3 per group. *p < 0.05, **p < 0.01, ***p < 0.001 compared to NT or EP only as appropriate unless specified.

To fully elucidate the role of electroporated BMDMs in anti-cancer immunity we subsequently examined the effect of treated BMDMs on cytotoxic CD8^+^ T cell responses. In contrast to CD4^+^ T cell activation, calcium EP was shown to increase the CD8^+^ T cell activation potential of melanoma conditioned BMDMs (Fig. 7a). EP had no effect on CD8^+^ T cell proliferation, however calcium increased activation in an apparently dose dependent manner (*p* = 0.0892). Intracellular granzyme B was detectable in both BMDMs and CD8^+^ T cells (Fig. 7b,c). BMDM calcium treatment resulted in an increase in granzyme B in both BMDMs and cocultured CD8^+^ T cells. EP decreased BMDM granzyme B levels (*p* < 0.05), which was also evident in decreased BMDM granzyme B levels in 500 μM calcium EP (ns) and 5 mM calcium EP (*p* = 0.05). BMDM EP had no effect on CD8^+^ T cell granzyme B levels across any of the tested conditions. Secretion of IL-10 and IFNγ were also measured as CD8^+^ T cells are significant sources of both IL-10 and IFNγ in vivo (Fig. 7d,e). Similarly to CD4^+^ T cell coculture, calcium induced a dose dependent increase in IL-10 levels, independently of EP, however calcium levels were of a 4 to sixfold lower concentration than from CD4^+^ T cell cocultures. No changes were observed in IFNγ secretion levels except by cocultures with BMDMs treated with 5 mM EP in which increased secretion was observed (*p* < 0.05).

## Discussion

ECT is a well-established treatment modality with high complete response rates. The EP pulses are optimized to result in reversible cell poration with minimal ablative effects. The cytotoxic effects of ECT are manifested by intracellular bleomycin during cell proliferation following treatment, as supported by the cell death shown above. Despite promising indications, calcium EP is not well established and is currently restricted to use on an investigative basis.

A significant proportion of the existing literature on electrochemotherapy and reversible electroporation in a cancer context use pulses with a voltage of 1200 V/cm. This is rationally designed to maintain consistency with clinically used pulsing parameters, which under ESOPE guidelines are 1000–1300 V/cm depending on the type of electrodes used. However, this voltage is ablative to both B16F10 cells and BMDMs, and thus a lower voltage is required to study the effect of reversible electroporation on these cells. In the pursuit of diligence, in the future it would be desirable to also consider the outcome of the experiments presented here using a pulse strength of 1200 V/cm, and to emphasize this discrepancy with existing literature in the context of the results presented. In vitro cell lines differ remarkably in the their ability to recover following the delivery of high voltage pulses, with anecdotal evidence suggesting smaller cells such as Lewis Lung Carcinoma cells require higher voltages to obtain effective reversible poration in comparison to larger cells such as B16F10 cells, it is still unclear if or how this translates to clinical efficacy^19^.

The fundamental mechanism of cell death following calcium EP is different to ECT as no chemotherapeutic is present. In vitro and murine preclinical observations have been supported by findings following veterinary use on equine sarcoids of the skin in which 14 of 16 treated tumours showed evidence of significant necrotic tumour cell death 1–3 weeks following treatment^31^. Furthermore, preliminary results of a single patient treated in a clinical colorectal cancer study have shown that treatment seems to be safe and treatment reduced obstruction of the colon^13^.

It has previously been shown that calcium EP results in necrotic cell death as a result of severe ATP depletion^27,32^. In agreement with this, here we have shown that the cell death following treatment is acute, in which cells do not regain membrane integrity or lose viability within 6 h of treatment. Cells which survive this initial insult show no apparent loss of proliferative potential.

Having successfully generated these treatment models in a melanoma cell line, we subsequently examined the effect of these on syngenic bone-marrow derived monocytes treated with B16F10 conditioned medium to mimic melanoma conditioning. These cells are susceptible to EP and regained membrane integrity following treatment. However, EP resulted in a subsequent reduction in viability after 24 h which was still evident 5 days after treatment. In tandem with this effect was a selective depletion of CD115^+^ F4/80^hi^ and CD206^+^ cells. CD115^+^ F4/80^hi^ and CD206^+^ are macrophage-like cells and also M2-like, in contrast to CD115^lo^ F4/80^mid^ cells which are monocyte-like and M1-like^29,33,34^. It is commonly considered that M2-like cells can support tumour growth and inhibit anti-cancer T cell responses while M1-like cells can exert inflammatory responses that can support anti-cancer immune responses^35^. While CD115^+^ F4/80^hi^ cells were depleted by EP, cell populations retained their arginase activity, indicating the retention an M2-like phenotype^36^. EP alone of BMDMs showed no subsequent effect on T cell proliferation or polarization as determine by surface markers but did result in increased IFNγ secretion in CD4^+^ T cell cocultures.

ECT did not induce any further cell death in comparison to EP alone in the first 24 h. By day 5 there is a further reduction in viability caused by ECT beyond the loss of viability seen with EP alone (*p* < 0.01). In a tumour context, there is a continual supply of TAMs from circulating monocytes and while it has been shown that some murine models contain populations of self-maintaining macrophage populations, it is likely that a continuous supply from the circulation is the predominant source of TAMs^37–39^. Therefore, while it is likely to effect coculture behaviour, it cannot be speculated whether viability declines seen after the first 24 h will have a meaningful effect on in vivo macrophage tumour biology. ECT followed the same trend of CD115^+^ F4/80^hi^ and CD206^+^ depletion as EP but did non-significantly decrease arginase activity indicating that ECT may impair M2-like responses.

Calcium EP of melanoma conditioned BMDMs displayed a remarkably different response to EP alone. Immediately following treatment cell size was significantly reduced. Following treatment an initial loss in viability was seen in cells treated with 5 mM calcium EP, however the scale of cell death in comparison to B16F10 cells was markedly reduced. At 1 h following treatment with 5 mM calcium there was a reduction of viability of 73 ± 3% of cells in B16F10s compared to only 23 ± 7% in melanoma conditioned BMDMs (p < 0.01 difference in means).

By 24 h calcium had played a protective role in improving viability following EP, and by day 5 no significant difference was observable between untreated cells and cells treated with calcium EP. It is unclear, how calcium can play a protective role which occurs following membrane resealing. Previously it has been shown that low calcium concentrations can aid pore resealing following EP^28^. However under the given conditions in which EP of BMDMs in calcium free conditions resulted in complete membrane resealing, this effect was not detectable. The protective role displayed here at 24 h is indicative of a protective role in cells which have already resealed^40,41^.

Calcium treatment, with or without EP reduced arginase activity. Calcium has been shown to influence purinergic receptor signalling in macrophages^42^. Over active ATPase activity generated by cells attempting to regain homeostatic intracellular calcium concentrations have been hypothesized as a potential cause of the severe depletion of ATP following calcium EP^43,44^. It is possible that calcium free buffers result in cytoplasmic calcium depletion which is energetically taxing on cells^45^. Further research is required to determine if low levels of calcium in EP buffers can reduce energetic stress on cells following treatment^46^.

Calcium EP diminishes the reduction of F4/80^hi^ CD115^+^ cells seen following EP despite the initial phenotypic shift towards cell shrinking, but instead of increasing arginase expression associated with these cells is responsible for a down regulation of arginase activity. This effect, with lower (500 μM) doses is independent of EP, indicating that passive calcium treatment can impart lasting effects on BMDMs. Mirroring this effect, calcium treatment of BMDMs in the absence of EP affected subsequent CD8^+^ T cell proliferation and both CD4^+^ and CD8^+^ T cocultures.

Cells were washed following treatment 3 times, negating the possibility of prolonged exposure to EP buffer or calcium treatments. However, calcium is known to interact with and affect the plasma membrane.

That calcium EP can upregulate IL-10 secretion from both CD4^+^ and CD8^+^ T cell cocultures in a dose dependent manner indicates that high dose calcium administration may inhibit anti-cancer immune responses as directed by exposed BMDMs^47^. However they exert complex effects, also increasing CD8^+^ T cell proliferation and granzyme B levels in activated T cells indicating the expansion of a cytotoxic immune response in a dose dependent manner^48,49^. Most strikingly, high dose (5 mM) calcium electroporated BMDMs were the only cells which displayed an increase in IFNγ from CD8^+^ T cell cocultures. IFNγ is a potent activator of Th1 anti-cancer immune responses^50^.

When administered in combination with EP, calcium dosage can affect the level of tumour cell death. From this data, it is clear that under the EP parameters used here, melanoma conditioned BMDMs are susceptible to poration by EP but relatively resistant to calcium EP induced cell death. However exposed cells are phenotypically and functionally altered. To our knowledge, this is one of the first studies examining the effect of reversible EP, as designed for the treatment of solid tumours, on immune cells, other than dendritic cells, which are also exposed to treatment. We conclude that BMDMs exposed to calcium EP maintain viability and promote cytotoxic T cell responses which may boost anti-cancer immune responses. Further investigation of other immune cell types, is required to determine if immune responses are compromised, and if therapeutic administration can be optimized to balance immunogenic tumour cell death with the retention and optimisation of the immune response by exposed immune cells.

## Materials and methods

### Cell line maintenance

The B16F10 cell line was purchased from and authenticated by the Developmental Therapeutics Program, Division of Cancer Treatment and Diagnosis Tumor Repository and regularly tested for the presence of mycoplasma. The B16F10 cell line was maintained in RPMI-1640 supplemented with 10% foetal calf serum (FCS) and 50 units/ml penicillin and 50 μg/ml streptomycin. Cells were cultured at 37 °C in a humidified chamber with 5% CO_2_. B16F10 cells were centrifuged at 200×*g* for 5 min during wash steps. For all washes, cells were centrifuged, then resuspended in respective buffers, centrifuged again, and resuspended as required.

### Ethical approval and ethical standards

All animal husbandry and handling was performed according to the Directive 2010/63/EU. Mice were culled specifically for use in this study under a euthanasia only licence, granted by the Animal Welfare Board of University College Cork, and was performed according to the Directive 2010/63/EU.

### Development and culturing of BMDMs

Animals were purchased from Envigo in the U.K. 4–6 week old female C57BL6J were euthanized by cervical dislocation. BMDMs were prepared as previously described^51^. Briefly, tibias and femurs were isolated and sterilized. The bone marrow was isolated and passed through a 70 μM filter. Red blood cells were lysed and remaining cells were cultured in high glucose DMEM with 1× Eagle′s minimum essential medium non-essential amino acids, β-mercaptoethanol (10 μM), sodium pyruvate (1 mM), FCS (10% v/v) and M-CSF (50 ng/ml, Biolegend) for 5 days. Cells were cultured for 5 days before adding 20× ultracentrifuged B16F10 conditioned medium to a final concentration of 1× for a further 24 h. Conditioned medium was prepared as previously described^51^, in brief 2.5 × 10^6^ B16F10 cells were incubated in a T175 flask in 20 ml RPMI supplemented with FCS (2% v/v) and P/S (1% v/v) for 48 h. Supernatant was isolated and ultracentrifuged in Vivaspin 20 tubes with a 3 kDa molecular weight cut off filter (GE Healthcare). Cells were isolated by gentle pipetting of EDTA (5 mM) in PBS following 5–15 min on ice. Bone marrow and BMDMs were centrifuged at 270 × *g* during wash steps.

### Reversible electroporation

1 × 10^6^ cells were washed and resuspended in HEPES EP buffer^52^ (10 mM HEPES, 250 mM sucrose, 1 mM MgCl_2_ in dH_2_0) with or without calcium (CaCl_2_ stock solution, Merck) at a final concentration of 500 μM, 2.5 mM, 5 mM or 10 mM or bleomycin (Bleomycin Teva, molarity was determined based on activity per mg and observation 1500 international units corresponds to 1 mg^53^) at final concentration of 10 nM in cuvettes with a 4 mm gap between two plate electrodes in a total volume of 800 μl. Reversible EP pulses were delivered by a square wave electroporator (BTX ECM 2001) with the following EP parameters: 8 pulses of 99 µs, 1 Hz, and 0.7 kV/cm (applied voltage to electrode distance ratio). Cells were rested for 20 min at 37 °C before further use.

### Clonogenic assay

Following treatment cells were washed twice and seeded in compete media. Seeding densities were empirically determine for each treatment regimen to ensure cells were 60–90% confluent after 24 h. After 24 h, to select for cells viable following treatment all non-adherent cells were discarded and adherent cells were isolated. Cells were then washed and seeded in 6 well plates in complete media in triplicate. The wells were checked every 2 days to ensure no acidification of the media had occurred. In instances where acidification of the media was apparent, 50% of the medium in all wells was replaced with fresh complete medium. Following 7–10 days, when wells containing untreated controls contained numerous well defined colonies of cells, the plates were stopped. To stop the plates, the medium was decanted and methanol was added to the wells for 5 min to fix adherent cells. The methanol was then decanted from the wells and Prodiff stain 2 was added to the wells for 5 min. Prodiff stain 2 was decanted from the plates and the plates were washed well under running tap water, ensuring not to dislodged fixed colonies. Plates were left to dry and then the fluorescence of the plate was then read using a Li-Cor plate reader (Odyssey) at 800 nm. Signal intensities were used to determine the level of colony growths in each well compared to untreated controls.

### Flow cytometry

Flow cytometry was performed using a 5 laser custom BD flow cytometer, cells were prepared and run at room temperature unless specified. Following addition of antibodies, where possible cells were kept in the dark.

To determine viability, 1 × 10^6^ cells were washed in 2 ml PBS. Propridium iodide (PI) (50 μg/ml) was added to cell suspension, vortexed and read within 60 s.

To determine pore formation by EP, propidium iodide (50 μg/ml) was added to cell solution (800 μl) directly prior to EP. Cells were not rested, and instead immediately transferred to polystyrene FACS tubes and washed in PBS. Cells were analysed immediately.

To determine surface markers, cells were washed in FACS buffer (PBS with FCS (2% v/v), sodium azide (0.01%) and EDTA (1 mM)). Cells were blocked in blocking solution (1 part FCS to 1 part FACS buffer) for 5 min. Cells were washed in FACS buffer and resuspended in a surface antibody cocktail for 30 min in the dark. Cells were washed twice before analysis. Surface stains used were CD3-APC (C363.29B, Novus-Bio), CD4-PE (RM 4–5, eBioscience), CD8-PE (53-6.7, Biolegend), F4/80-PE (BM8, Biolegend), CD11b-BV605 (M1/70, BD Biosciences), CD115-PE-Cy7 (AFS98, Invitrogen), CD206-PerCP-Cy5 (C068C2, Biolegend).

For granzyme B staining, surface markers were stained first. Cells were then fixed in 2% paraformaldehyde for 20 min. Cells were washed 3 times in Intracellular staining permeabilisation wash buffer (Biolegend) and granzyme B-PE-Cy7 antibody (QA16A02, Biolegend) was then added for 30 min. Cells were washed twice in FACS buffer.

### Arginase assay

BMDMs were washed twice and lysed in Tris–HCl (10 mM, pH7.4) with protease inhibitors and Triton X-100 (1% v/v) for 30 min on ice with vigorous vortexing every 10 min. The samples were centrifuged at 13,000×*g* for 20 min to remove debris. Protein content was normalized. Arginase activity was assayed using an Arginase Activity Assay Kit (Sigma). Briefly, cell lysate (40 μl) was added to each well in quadruplicate. Substrate buffer (10 μl) was added to each sample well and each sample blank well received no addition. Plates were sealed and incubated at 37 °C for 2 h. Urea reagent (200 μl) was added to all wells to stop the reaction and substrate buffer (10 μl) was added to the sample blank wells. Plates were incubated at RT for 60 min. The absorbance was read at 430 nm using a SpectraMax M2 (Molecular Devices).

### CD4^+^ and CD8^+^ T cell isolation, CFSE staining and coculture

BMDMs were treated, washed 3 times and cultured for 24hrs. Spleens were isolated from syngenic mice and passed through a 70 μm filter. RBCs were lysed in lysis buffer and CD4^+^ or CD8^+^ T cells were isolated using negative selection magnetic bead labelling kits (Miltenyi Biotec) according to the manufacturer’s instructions. Separation was confirmed by analysis of CD4 and CD8 staining by flow cytometry. All cells were confirmed to be > 85% purity prior to use. Separated T cells were labelled with CFSE. The reaction was stopped by adding 10 volumes of complete medium. Cells were washed twice following stimulation with conditioned medium or cytokines and counted before being added to cocultures. 10^5^ T cells and 10^5^ BMDMs were seeded in 96 well suspension plates in 200 μl and incubated for 5–7 days. Medium colour was observed daily to ensure no acidification of wells occurred.

### ELISA

IL-4, IL-10 and IFNγ Mouse ELISA Max™ Deluxe Sets (Biolegend) were used according to the manufacturer’s instructions. Briefly, plates were blocked overnight at 4 °C in capture antibody diluted in coating buffer, all subsequent steps were performed at room temperature. Plates were emptied and washed. For each wash plates were emptied, filled with PBS + 0.05% tween and allowed soak for 30 s 3 times. The plates were then blocked for 1 h in assay diluent (from kit) with shaking. Plates were washed and samples, standards and controls were added to the plates for 2 h with shaking. Plates were washed and detection antibody was added for 60 min with shaking. The plates were washed and diluted avidin-HRP was added for 30 min with shaking. The plates were washed and freshly made substrate solution was added. The plates were incubated in the dark until a visible colour change had occurred. The reaction was stopped by adding an equal volume of 1 M HCl and the plates were promptly read at 450 and 570 nm. 570 nm readings were subtracted from 450 nm readings before analysis.

### Statistical analysis

Statistical analysis was performed using paired and un-paired Student’s t-test for differences between groups. Flow cytometry data was analysed using FlowJo version 10.5.3. Statistical analysis was performed using Graphpad Prism version 5.03.

## Data Availability

All data generated or analysed during this study are included in this published article.
